# Increasing utilization of Internet-based resources following efforts to promote evidence-based medicine: a national study in Taiwan

**DOI:** 10.1186/1472-6947-13-4

**Published:** 2013-01-07

**Authors:** Yi-Hao Weng, Ken N Kuo, Chun-Yuh Yang, Heng-Lien Lo, Ya-Hui Shih, Chiehfeng Chen, Ya-Wen Chiu

**Affiliations:** 1 Department of Pediatrics, Chang Gung Memorial Hospital, Chang Gung University College of Medicine, Taipei, Taiwan; 2 Center for Evidence-Based Medicine, College of Medicine, Taipei Medical University, Taipei, Taiwan; 3 Division of Preventive Medicine and Health Services Research, Institute of Population Health Sciences, National Health Research Institutes, Miaoli, Taiwan; 4 Department of Public Health, Kaohsiung Medical University, Kaohsiung, Taiwan; 5 Master Program in Global Health and Development, College of Public Health and Nutrition, Taipei Medical University, Taipei, Taiwan; 6 Division of Plastic Surgery, Department of Surgery, Taipei Medical University Wan Fang Hospital, Taipei, Taiwan

## Abstract

**Background:**

Since the beginning of 2007, the National Health Research Institutes has been promoting the dissemination of evidence-based medicine (EBM). The current study examined longitudinal trends of behaviors in how hospital-based physicians and nurses have searched for medical information during the spread of EBM.

**Methods:**

Cross-sectional postal questionnaire surveys were conducted in nationally representative regional hospitals of Taiwan thrice in 2007, 2009, and 2011. Demographic data were gathered concerning gender, age, working experience, teaching appointment, academic degree, and administrative position. Linear and logistic regression models were used to examine predictors and changes over time.

**Results:**

Data from physicians and nurses were collected in 2007 (n = 1156), 2009 (n = 2975), and 2011 (n = 3999). There were significant increases in the use of four Internet-based resources – Web portals, online databases, electronic journals, and electronic books – across the three survey years among physicians and nurses (*p* < 0.001). Access to textbooks and printed journals, however, did not change over the 4-year study period. In addition, there were significant relationships between the usage of Internet-based resources and users’ characteristics. Age and faculty position were important predictors in relation to the usage among physicians and nurses, while academic degree served as a critical factor among nurses only.

**Conclusions:**

Physicians and nurses used a variety of sources to look for medical information. There was a steady increase in use of Internet-based resources during the diffusion period of EBM. The findings highlight the importance of the Internet as a prominent source of medical information for main healthcare professionals.

## Background

Physicians and nurses are main healthcare professionals in clinical service. They should periodically update their medical information to optimize patient care and outcome [1]. In the past, knowledge was obtained from textbooks, colleagues, and journal articles [2,3]. In recent years, development of medical informatics has provided an opportunity for easy access to up-to-date medical information. The Internet is widely used for locating medical knowledge now [4,5].

There are a variety of Internet-based resources, such as Web portals, online databases, electronic journals, and electronic books [6-8]. Web Portals have been a good starting point to search medical information. A growing trend of using Web portals has been noticed around the world [1]. However, challenges exist regarding how to easily find quality information from an overwhelmingly large number of Web-search results. In addition, the publication policy of traditional journals and textbooks has been changed with the advent of Internet [9]. The number of medical journals and textbooks that offer electronic versions of their publications has grown tremendously [10]. There is an increase of free available electronic journals and books. Furthermore, online databases offer access to retrieve the summarized evidence-based information. Mastery of access to online databases has become pertinent for improving the quality of clinical practice [11-15].

Policymakers of healthcare institutions have implemented information systems to support healthcare professionals’ use of evidence-based medicine (EBM), which involving free access to Internet-based EBM resources [16]. They need to know whether these resources are regularly being employed to apply appropriate medical information on patient care. There are increasing studies investigating the information-searching behavior of health professionals [17,18]. However, very few have tracked the trends in search of medical information over time.

Since the beginning of 2007, the National Health Research Institutes (NHRI) has overseen a collaborative project to provide information systems in support of clinical practice with evidence for regional hospitals of Taiwan [19,20]. It is unclear exactly how such implementation is influencing the ways in which healthcare professionals obtain medical information. Thus, we examined responses to three questionnaire surveys administered between 2007 and 2011. The purpose of this was to demonstrate the longitudinal trends in behavior patterns among hospital-based physicians and nurses in searching medical information during a 4-year promotional period.

## Methods

### Initiatives of NHRI

The project launched by NHRI to promote the implementation of EBM was multifaceted, consisting of offering information resources and carrying out a series of activities. Overall, the program set up a Chinese EBM Web site, offered free usage of the Cochrane Library, and provided Chinese abstracts of the Cochrane Database of Systematic Reviews for health professionals to access. Furthermore, NHRI conducted monthly workshops with a total of 4714 attendee throughout Taiwan. In addition, this campaign established the Taiwan Evidence-Based Medicine Association (TEBMA) in 2007. In cooperation with TEBMA, NHRI organized annual conferences and competitions pertaining to EBM implementation.

## Questionnaire surveys

### Design

NHRI developed a structured questionnaire using questions based on previous literature [3,21-23]. The first survey was conducted at the beginning of 2007. The second and third surveys were conducted in 2009 and 2011, respectively.

### Samplings

The targets in this study were physicians and nurses working in Taiwan’s regional hospitals. A regional hospital is defined as a secondary care hospital, as appraised by Taiwan’s Joint Commission of Hospital Accreditation. Sixty-one regional hospitals, including a total of approximately 5000 physicians and 20000 nurses, were enrolled. Only the 4 regional hospitals that did not have a collaborative project with NHRI to promote the implementation of EBM were not enrolled. In the 2007 survey, the postal questionnaires were distributed at random to 30 members of the clinical staff (with the title of either medical doctor or nurse) of each regional hospital [19]. In order to obtain more questionnaires from nurses a cluster sampling design was used in the 2009 survey, as described previously [11]. Briefly, questionnaires were distributed to all staff at 13 hospitals selected at random, including 4 located in northern Taiwan, 3 in eastern Taiwan, 3 in southern Taiwan, and 3 in central Taiwan. Since we achieved a higher number of respondents using the cluster sampling method in the 2009 survey compared to the earlier survey, we continued the same cluster sampling method for our subsequent survey in 2011. All questionnaire returns were anonymous.

### Survey questions

The survey included items for measuring the usage patterns of 7 informational resources: (1) Web portals (e.g., Google, Yahoo), (2) online databases, (3) electronic textbooks, (4) electronic journals, (5) textbooks, (6) printed journals, and (7) colleague consultations. The frequency was classified by Likert’s 4-point scale (always, often, seldom, and never).

The survey further explored the usage of access to 9 online databases: 2 databases in Chinese and 7 databases in English. Chinese databases included the Index to Chinese Periodical Literature (ICPL) and the Chinese Electronic Periodical Service (CEPS). English databases included the Cumulative Index to Nursing & Allied Health Literature (CINAHL), the Cochrane Library, MD Consult, MEDLINE/PubMed, ProQuest, UpToDate, and Micromedex. Utilization was defined as access at least once per month during the previous 6 months.

The background characteristics — including gender, age, faculty position, director position, working experience, EBM training, and academic level — were also examined. Age of the respondents was classified into four sections: 20–30, 31–40, 41–50, and more than 50 years old. EBM training was defined as participation in the EBM workshops or related curricula. In addition, academic level was divided into five categories: (1) those with a diploma from a technical school; (2) those who graduated from a junior college (2-year university); (3) those with a bachelor’s degree (The curriculum is 7 years for medical school, 6 years for dental school and 4 years for nursing school); (4) those with a master’s degree; and (5) those with a doctoral degree.

### Validity and reliability

Content validity of the questionnaire was examined by 9 experts with more than 15 years each of professional clinical service. The internal consistency of all indexes was estimated using Cronbach’s coefficient alpha. The reliability and validity of the current survey were well supported by sufficient content validity index and Cronbach’s coefficient alpha [20].

#### Ethical considerations

The Ethical Review Board of NHRI approved the study protocol. The questionnaire was accompanied an introductory letter stating the purpose of this study and promising confidentiality. Returns were considered as indicating consent to participate in this study.

#### Statistical analyses

Likert’s 4-point scale for the use of informational resources was dichotomized for further analyses. A self-rating report of “always” or “often” was regarded as a favorable answer. The other two (“seldom” and “never”) were viewed as unfavorable answers. The statistical analyses were conducted using a commercially available program (SPSS 12.0 for Windows, SPSS Inc., Illinois, USA). Pearson’s chi-square test was used to compare the differences. Logistic regression was used to examine relationships among variables. Significance was defined as *p* < 0.05. All odds ratio (OR) and 95% confidence intervals (CI) were adjusted for the demographic characteristics and survey years.

## Results

### Demographic data of respondents

This study enrolled 1156 participants in 2007 (605 physicians and 551 nurses), 2975 in 2009 (563 physicians and 2412 nurses), and 3999 in 2011 (645 physicians and 3354 nurses). The valid response rate was 63.2% in 2007, 57.8% in 2009, and 58.2% in 2011. Their demographic characteristics are shown in Table 1.

**Table 1:** Demographic characteristics of participants

**Profession**	**Physician**			**Nurse**		
**Year**	**2007**	**2009**	**2011**	**2007**	**2009**	**2011**
number	605	563	645	551	2412	3354
**Gender (%)**						
Male	88.1	82.6	81.4	2.2	0.7	1.1
Female	11.9	17.4	18.6	97.8	99.3	98.9
**Age (y) (%)**						
20–30	12.3	19.8	12.4	42.1	42.0	49.4
31–40	43.8	41.8	50.2	42.8	44.1	41.5
41–50	33.6	25.4	25.0	13.5	12.5	8.3
> 50	10.3	13.0	12.4	1.6	1.4	0.8
**Working period (y) (%)**						
< 5	25.8	31.2	25.2	19.9	18.2	31.5
5–10	25.6	29.6	35.7	36.4	43.3	39.8
> 10	48.6	39.2	39.1	43.8	38.5	28.7
**Academic level (%)**						
Technical school	0	0	0	40.5	53.1	43.5
Junior college	0	0	0	31.8	26.2	33.0
Bachelor’s*	80.6	80.2	78.3	24.3	18.0	21.2
Master’s	15.5	15.6	16.0	3.4	2.6	2.2
Doctorate	3.9	4.2	5.7	0	0.1	0.1
**EBM training (%)**	55.2	50.4	39.5	22.7	14.3	15.6
**Director (%)**	34.3	24.8	25.4	22.1	11.8	8.9
**Faculty (%)**	33.9	38.8	39.9	23.4	14.7	14.5

*Bachelor’s curriculum is 7 years for medical school, 6 years for dental school, and 4 years for nursing school.

There were significant differences in the demographic characteristics between physicians and nurses. Males were more common among physicians; females were more common among nurses. Most physicians were between the ages of 30 and 50 years old, while most nurses were 20–40 years old. Most physicians had at least a bachelor’s degree; but most nurses didn’t. Physicians more often received EBM training than nurses did (*p* < 0.001). Administrative and faculty positions were more common among physicians than nurses.

### Resources of medical information

**Figure 1:**
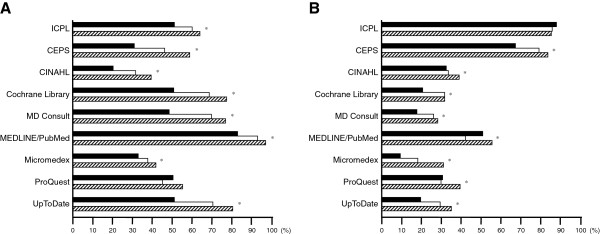
**Resources of information searching.** (**A**) Physicians; (**B**) Nurses. Solid bar: 2007; empty bar: 2009; hatched bar: 2011. * *p* < 0.05 among the three groups.

The information-searching patterns of physicians and nurses are illustrated in Figure 1. The most common source of medical information in all survey years for physicians was Web portals (77.9%, 84.6%, and 95.1% in 2007, 2009, and 2011, respectively). As for nurses, the most common resource in 2007 was textbooks (73.2%) but was Web portals in 2009 (77.8%) and 2011 (94.2%). There were significant increases in the usage of four Internet-based resources — including Web portals, online databases, electronic journals, and electronic books – across the three survey years among physicians and nurses. In addition, there was no significant change in the utilization of textbooks and printed journals among physicians and nurses. Furthermore, there was a significant increase of colleague consultations in 2011 than in 2007 and 2009 among nurses, but not among physicians.

### Demographic predictors of usage of Internet-based resources

Demographic data were incorporated into logistic regression analysis to determine the relationship of the usage of each Internet-based information source (Table 2). There were significant relationships between the usage of Internet-based resources and users’ characteristics, including age, academic degree, working experience, and director and faculty positions. The use of every Internet-based source significantly increased from 2007 to 2011 (*p* < 0.001). Male physicians were more likely to seek medical information through Web portals than female physicians (*p* < 0.01). In addition, young physicians (age ≤ 50) tended to access the online databases (*p* < 0.01) and electronic journals (*p* < 0.05) more than elder physicians (age  > 50). Young nurses (age ≤ 30) were more likely to access every Internet-based resource than elder nurses (age  > 50) (*p* < 0.05). Furthermore, physicians with a doctoral degree were more likely to use electronic journals than physicians without an advanced degree (*p* < 0.01). Nurses with a doctoral degree were more likely than those with a bachelor’s degree to use all 4 Internet-based resources (*p* < 0.05). Nurses with a master’s degree were more likely to access Web portals, online databases, and electronic journals than those with a bachelor’s degree (*p* < 0.05). In contrast, nurses with a degree from a technical school were less likely to use Web portals, online databases, and electronic journals than those with a bachelor’s degree (*p* < 0.001). Faculty physicians tended to access the online databases and electronic journals more than physicians without a faculty position. Faculty nurses were more likely to access Web portals, online databases, and electronic journals than nurses without a faculty position. Physicians with EBM training were more likely to access the online database than those without EBM training. Physicians with a working period less than 5 years were less likely to access Web portals than physicians with a working period greater than 10 years.

**Table 2:** Demographic predictors of access to Internet-based resources by multivariate logistic regression analysis, OR (95% CI)

**Profession**	**Physician (n = 1813)**				**Nurse (n = 6317)**			
**Resource**	**Web portals**	**Online databases**	**Electronic journals**	**Electronic books**	**Web portals**	**Online databases**	**Electronic journals**	**Electronic books**
**Year (2007, reference)**								
2011	5.00*** (3.21–7.79)	6.14*** (3.96–9.52)	3.32*** (2.32–4.74)	2.29*** (1.77–2.95)	13.1*** (9.09–19.0)	4.83*** (3.57–6.54)	7.32*** (5.37–9.98)	4.82*** (3.57–6.52)
2009	1.51* (1.07–2.17)	1.49* (1.08–2.07)	NS	NS	2.33*** (1.66–3.29)	NS	NS	NS
**Gender (female, reference)**								
Male	0.44** (0.24–0.81)	NS	NS	NS	NS	NS	NS	NS
**Age ( > 50 y, reference)**								
20–30	NS	4.65*** (2.15–10.1)	2.19* (1.11–4.31)	NS	2.19* (1.06–4.55)	2.34** (1.25–4.36)	2.83** (1.50–5.33)	2.39** (1.25–4.59)
31–40	NS	3.97*** (2.21–7.13)	1.91* (1.13–3.22)	NS	NS	1.85* (1.02–3.36)	NS	NS
41–50	NS	2.22** (1.40–3.52)	1.58* (1.03–2.43)	NS	NS	NS	NS	NS
**Academic (college, reference)**								
Ph.D.	NS	NS	3.24** (1.33–7.85)	NS	1.94* (1.01–3.88)	10.3*** (4.42–24.0)	6.68*** (2.98–15.0)	2.25** (1.26–4.00)
Master’s	NS	NS	NS	NS	1.51* (1.01–2.26)	2.21*** (1.59–3.08)	1.58** (1.12–2.22)	NS
Junior college	—	—	—	—	NS	NS	NS	NS
Technical school	—	—	—	—	0.60*** (0.47–0.77)	0.68*** (0.57–0.80)	0.68*** (0.57–0.81)	NS
**Director (no, reference)**								
Yes	1.78** (1.21–2.61)	NS	NS	NS	NS	NS	1.62*** (1.30–2.03)	NS
**EBM training (no, reference)**								
Yes	NS	1.44* (1.07–1.93)	NS	NS	NS	NS	NS	NS
**Faculty (no, reference)**								
Yes	NS	2.44*** (1.67–3.56)	1.99*** (1.45–2.75)	NS	1.67*** (1.27–2.18)	1.50*** (1.24–1.80)	1.56*** (1.28–1.91)	NS
**Working period ( >10 y, reference)**								
< 5	0.39** (0.20–0.74)	NS	NS	NS	NS	NS	NS	NS
5–10	NS	NS	NS	NS	NS	NS	NS	NS

NS: no significance; * *p* < 0.05; ** *p* < 0.01; *** *p* < 0.001.

### Trends in the use of 9 online databases

**Figure 2:**
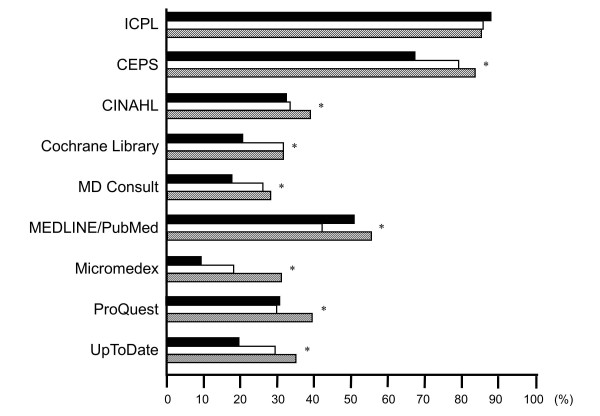
**Usage of online databases.** (**A**) Physicians; (**B**) Nurses. Solid bar: 2007; empty bar: 2009; hatched bar: 2011. * *p* < 0.05 among the three groups. ICPL: the Index to Chinese Periodical Literature. CEPS: the Chinese Electronic Periodical Service. CINAHL: the Cumulative Index to Nursing & Allied Health Literature.

Participants were asked to report how often they accessed the online databases. Figure 2 displays the changes in the use of 9 online databases for the three survey years. Among physicians there were significant increases in the usage of 8 online databases — ICPL, CEPS, CINAHL, the Cochrane Library, MD Consult, MEDLINE/PubMed, Micromedex, and UpToDate — across the three survey years. In addition, there were significant increases in the usage of 8 online databases — CEPS, CINAHL, the Cochrane Library, MD Consult, MEDLINE/PubMed, Micromedex, ProQuest, and UpToDate — over the 4-year study period for nurses.

## Discussion

The current evaluation has illustrated how information-searching behaviors changed among main healthcare professionals. We characterized trends in the search of medical information across the nationwide regional hospitals of Taiwan, using data from 3 national surveys conducted with main healthcare professionals in 2007, 2009, and 2011. Coupled with these three surveys we showed a growth in the use of Internet-based resources. The data suggest that there has been a sustained effect following the dissemination of EBM implementation during the 4-year study period,

To our knowledge, this study is the first to examine national trends in sources of medical information. The results highlight that the Internet has become an increasingly important source of medical information, which is consistent with recent research findings indicating that the Internet has become the most common source of medical information [3,24]. Internet-based resources can provide easy access to reduce barriers, such as physical distance from a library. In our study, medical information in which main healthcare professionals looked for has shifted to electronic media gradually.

Our study demonstrated a high level of usage with the Web portals. The use of Web portals increased steadily across the 3 survey years. Searching for medical information through Web portals has both advantages and disadvantages. Although Web portals are a convenient and easily accessible source of medical information, challenges regarding the quality and credibility of medical information exist. With the advent of Web portals (such as the development of Google Scholar), the quality of searching has been improving [25,26]. Nevertheless, online databases are still more practical for retrieving evidence-based search results than Web portals [26]. Familiarity with evidence-based sites is essential for healthcare providers in order to improve the quality of medical care. Our previous study showed increasing access of online evidence retrieval systems among physicians and nurses after a 2-year promotion of EBM [11]. The current survey has further extended the inquiry by demonstrating a sustaining increase in the utilization of four Internet-based resources over 4-year period. We found that not only increased usage of Internet-based resources in general, but also the trend applied to all 8 popular online EBM databases. During our study period, NHRI offered free access to the Cochrane Library to promote EBM dissemination [11,27]. Nevertheless, both physicians and nurses used a variety of databases to look for medical information rather than using the Cochrane Library alone. In addition, our study demonstrated that the increased usage of online database was proportional to that of Web portals. We found the majority of physicians and nurses used the online database for self-learning or clinical practice [28,29]. However, we did not know why they accessed the Web portals. Further studies are needed to verify the difference in the motivation of usage between online database and Web portals.

In our study population, electronic journals have replaced printed journals as the most commonly used resource when looking for medical information. With the advance of medical informatics, the number of electronic books and journals is increasing exponentially. In addition, access to Web portals and online databases can enhance the utilization of electronic journals and electronic books. Folb *et al.* reported that healthcare professionals regarded convenience as the most important factor for accessing printed or electronic formats at times of need [7]. These data suggest electronic resources have become more important for healthcare professionals when searching for medical information.

Differences in the backgrounds between physicians and nurses may lead to different behaviors of information searching through the Internet. In this study, both groups were increasingly using the Internet to find answers to clinical questions. However, their demographic predictors of access to Internet-based resources were somewhat different. First, gender and working period were significant factors in relation to the usage of Web portals among physicians only, while in nursing profession, it was not significant. Second, academic degree carried more significance in nurses than in physicians. Third, age and faculty position were important factors of usage for both physicians and nurses; this finding is in accordance with previous studies [20,30]. And, fourth, electronic books were less associated with the personal characteristics which was similar to a report showing that use of electronic books was not related to users’ personal profiles [7]. By distinguishing these characteristics, educational providers could devise better strategies to enhance their utilization. For example, physicians and nurses with senior age or without faculty position need more educational programs and interventions to prompt online utilization. In our study, the other personal characteristics – such as gender and working period – carried less significant influence in the usage. These results can provide stakeholders and promoters with valuable information to increase access to evidence-based information.

This study has several limitations common to survey research, such as potential biases from the incomplete response and self-reported method. We cannot be sure that these self-reported changes were fully translated into actual behaviors. In addition, some methodological issues should be considered in this study. First, we modified the sampling method in the 2009 and 2011 surveys to collect more questionnaires from nurses. Nevertheless, we believe the influence of this alteration was minimal because of high similarity of demographic backgrounds across the three questionnaire surveys. Second, our study did not take other factors which have enabled Internet health information searching into account. For instance, the influences of institutional characteristics on the participants, such as their own organizational change, were not determined. Third, this study did not examine the impact of such changes on clinical practice. Further studies are necessary to determine the usage of Internet-related resources in the improvement of patient outcome.

## Conclusion

In conclusion, this study has provided nationally representative estimates for the preferences of main healthcare professionals in searching for medical information. We tracked trends in informational resources sought by main healthcare professionals over time. Our data indicated that the Internet was increasingly being used by physicians and nurses as main source of medical information in Taiwan. The results portray a tectonic shift in the ways in which main healthcare practitioners searched for the medical information, with increasing access of information through Internet-based resources among healthcare providers.

## Competing interests

The authors declare that they have no competing interests.

## Authors’ contributions

YHW and YWC conceived and developed the study and drafted the manuscript. KNK was responsible for organizing the team, obtaining funding and drafting the manuscript. CYY, HLL, YHS, and CC assisted with coordinating the study and drafting the manuscript. All authors read and approved the final manuscript.

## Pre-publication history

The pre-publication history for this paper can be accessed here:

http://www.biomedcentral.com/1472-6947/13/4/prepub
